# Cancer patients as frequent attenders in emergency departments: A national cohort study

**DOI:** 10.1002/cam4.1728

**Published:** 2018-08-17

**Authors:** Ting Hway Wong, Zheng Yi Lau, Whee Sze Ong, Kelvin Bryan Tan, Yu Jie Wong, Mohamad Farid, Melissa Ching Ching Teo, Alethea Chung Pheng Yee, Hai V. Nguyen, Marcus Eng Hock Ong, N. Gopalakrishna Iyer

**Affiliations:** ^1^ Singapore General Hospital Singapore Singapore; ^2^ Duke‐National University of Singapore Medical School Singapore Singapore; ^3^ Policy Research and Evaluation Division Ministry of Health Singapore Singapore; ^4^ National Cancer Centre Singapore Singapore; ^5^ Saw Swee Hock School of Public Health Singapore Singapore; ^6^ School of Pharmacy Memorial University of Newfoundland St John's Newfoundland Canada

**Keywords:** access, cancer, emergency, frequent attenders, healthcare utilization

## Abstract

**Background:**

Cancer patients contribute significantly to emergency department (ED) utilization. The objective of this study was to identify factors associated with patients becoming ED frequent attenders (FA) after a cancer‐related hospitalization.

**Methods:**

A retrospective cohort study was conducted using national administrative, billing, and death records of Singapore residents discharged alive from Singapore public hospitals from January 2012 to December 2015, with a primary discharge diagnosis of cancer. Patients with four or more ED visits within any 12‐month period after discharge from their index hospitalization were classified as FA. Time to FA distribution was estimated using the Kaplan‐Meier method, and factors associated with risk of FA were identified using multivariate Cox regression analyses.

**Results:**

Records for 47 235 patients were analyzed, of whom 2980 patients were FA within the study period. Age (<17 years, hazard ratio [HR] 2.92, 95% CI 2.28‐3.74; 75‐84 years, HR 1.29, 95% CI 1.16‐1.45; and ≥85 years, HR 1.71, 95% CI 1.45‐2.02, relative to age 55‐64), male gender (HR 1.26, 95% CI 1.16‐1.37), Charlson comorbidity index (HR 1.21, 95% CI 1.19‐1.23), and socioeconomic factors (Medifund use, HR 1.40, 95% CI 1.23‐1.59; housing subsidy type, HR 2.12, 95% CI 1.77‐2.54) were associated with increased risk of FA. Primary malignancies associated with FA included brain and spine (HR 2.51, 95% CI 1.67‐3.75), head and neck cancers (tongue, HR 2.05, 95% CI 1.27‐3.31; hypopharynx, HR 2.72, 95% CI 1.56‐4.74), lung (trachea and lung, HR 1.57, 95% CI 1.13‐2.18; pleural, HR 3.69, 95% CI 2.12‐6.34), upper gastrointestinal (stomach, HR 1.93, 95% CI 1.26‐2.74; esophagus, HR 4.13, 95% CI 2.78‐6.13), hepato‐pancreato‐biliary (liver, HR 1.42, 95% CI 1.01‐2.00, pancreas, HR 2.48, 95% CI 1.72‐3.59), and certain hematological malignancies (diffuse non‐Hodgkin's lymphoma, HR1.59, 95% CI 1.08‐2.33, lymphoid leukemia, HR 1.86, 95% CI 1.21‐2.86). Brain (HR 1.69, 95% CI 1.27‐2.26), lung (HR 1.31, 95% CI 1.01‐1.71), liver (HR 1.46, 95% CI 1.14‐1.89), and bone (HR 1.35, 95% CI 1.04‐1.76) metastases were also associated with FA.

**Conclusion:**

There are cancer‐specific factors contributing to ED frequent attendance. Additional resources should be allocated to support high‐risk groups and prevent unnecessary ED use.

## 1 INTRODUCTION

Cancer patients contribute to a high proportion of emergency department (ED) utilization.^1^, ^2^ A recent study of ED utilization by cancer patients showed that the commonest ED diagnoses were similar to those of the general population (pneumonia, chest pain, and urinary tract infection).^2^ Nevertheless, many cancer patients attend ED for issues unique to their diagnoses, stage, or treatment. Hence, several studies have focused on certain subgroups, such as end‐of‐life patients,^3^, ^4^, ^5^, ^6^, ^7^, ^8^ and patients suffering from complications or side effects of chemotherapy, surgery, or radiotherapy.^9^, ^10^, ^11^, ^12^, ^13^

A smaller cohort of cancer patients return to the ED multiple times, and become ED frequent attenders (FA), commonly defined as patients making four or more visits within a 12‐month period.^14^, ^15^, ^16^, ^17^, ^18^ Studies that examine general ED patients have shown that FAs have a higher chronic disease burden, different socioeconomic profiles,^14^, ^17^, ^18^, ^19^, ^20^ and higher utilization of nonemergent healthcare services.^15^, ^16^ Yet, little is known about cancer patients who become FAs.

We hypothesized that some cancer patients were at higher risk of becoming FAs due to disease‐specific symptomology and oncologic management. Defining cancer‐specific risk factors for FA would be critical in the identification of patients with unmet needs. This could present opportunities for improving the quality of cancer care, both in the ED itself,^21^ as well as in the community, with the ultimate goal of reducing the need for ED visits.^2^ Therefore, the primary objective of this study was to identify risk factors for FA by cancer patients using a national database of all patients treated in public sector hospitals. The secondary objective was to examine the trajectory of FA patients after they became FA, including their subsequent survival, and time to repeat FA. Survival from FA would clarify whether the ED visits were mostly at the end‐of‐life phase of the disease, while repeat FA would suggest that these patients continued to have high ED utilization and unmet needs.

## 2 METHODS

### 2.1 Study design, setting, data sources, and participants

This national retrospective cohort study was conducted using administrative data of ED visits, inpatient admissions, and financial claims from the Ministry of Health.^22^ Inclusion criteria were as follows: Singapore residents, alive at discharge, primary discharge diagnosis of cancer by International Classification of Diseases codes (ICD‐10‐AM: C00 ‐ C96), from January 2012 to December 2015. Death data as of 31 December 2015 were obtained from the Singapore Registry of Births and Deaths.

### 2.2 Variables

Demographic information (age, gender, ethnicity) was obtained from claims data.

Socioeconomic variables were derived from mapping residential postal codes to housing type,^23^ eligibility for subsidized primary care under the Community Health Assist Scheme (CHAS),^22^ and receiving financial assistance from the government Medifund^22^ scheme for the index hospitalization.

The first hospitalization during the study period with a primary diagnosis of cancer was considered the index hospitalization. Clinical information was extracted from discharge diagnoses (cancer sites, comorbidities) and admission record (length of stay, discharge destination) for the index hospitalization. Patients were designated as FA once they made four or more ED visits within any 12‐month period during the study period after discharge from the index hospitalization. The outcome variable, time to FA, was the time from discharge from index hospitalization to meeting FA criteria. Patients who died without meeting FA criteria were censored at their date of death, while those who were still alive at the end of the study period were censored as at 31 December 2015. Patients who died after fulfilling FA criteria were still considered FA and not censored.

Primary cancer sites were grouped by two‐digit ICD‐10‐AM codes according to the US National Cancer Institute Surveillance, Epidemiology, and End Results (SEER) codes for cancers deemed to be single‐site primaries.^24^ Metastases were grouped by three‐digit codes into brain, bone, lymph node, lung, liver, other gastrointestinal, and other metastases. Primary cancer sites with low event rates (fewer than ten patients becoming FA) were regrouped (Table S1).

### 2.3 Statistical analysis

Time to FA distribution was estimated using Kaplan‐Meier method, and factors associated with risk of FA were identified using multivariable Cox regression analyses. The following variables were examined in the main Cox model: age at index admission, gender, Charlson comorbidity index (excluding cancer variables, unadjusted for age), primary and metastatic sites of cancer, length of stay, discharge destination, and socioeconomic variables.

Additional sensitivity analyses of the main Cox model were performed: (a) Cancer sites classified based on original ICD‐10‐AM two‐digit codes (without regrouping according to SEER codes, in the event that sites that may be biologically similar but cause different symptoms); (b) cancer sites classified by SEER groups, with low event rate primary sites all grouped into a single category “others” (to examine the cancer primary sites without any attempt at forced grouping of similar “rare” primary sites); (c) excluding patients with missing ethnicity and/or housing type; (d) including interaction terms for ethnicity and all three socioeconomic indicators (Medifund use, income percentile, and housing type); (e) excluding patients aged <17 years (to examine the adult population without the effect of pediatric‐dominant tumors); and (f) addition of interaction terms for age‐group and the cancer types known to be more common in pediatric and young adult age‐groups.

For the secondary analysis of the trajectory of FA patients, survival was measured, from the day the patient first met FA criteria to the day of death. To examine patients with continued high unmet needs after becoming FA, ED visits were examined for the subsequent 12‐month period after the patient first met FA criteria. Time to repeat FA was measured, from the day the patient first met FA criteria to the day the patient made an additional four or more ED visits.

STATA (version 13.0, StataCorp. 2013. Stata Statistical Software: Release 13. College Station, TX: StataCorp LP.) was used to perform statistical analyses, and a two‐sided *P*‐value < 0.05 was considered statistically significant.

### 2.4 Missing data

The only variables with missing data in this study were housing type (7149, 15.1%) and ethnicity (5971, 12.6%). Impact of missing data on the identification of risk factors for FA was evaluated via sensitivity analysis set (3). No imputation was performed.

### 2.5 Potential bias

As this study was limited to public hospitals in Singapore, we could not capture the private hospital admissions and ED visits. Hence, some of the non‐FAs in this study, especially patients with better insurance coverage, may have in fact been FAs, if private hospital ED visits had been captured. However, the majority of health care by Singapore residents is provided by public hospitals^22^; hence, we expect this effect to be minimal.

### 2.6 Ethical approval

The first author's (Singapore General Hospital) Institutional Review Board granted ethical approval for this study.

## 3 RESULTS

**Figure 1:**
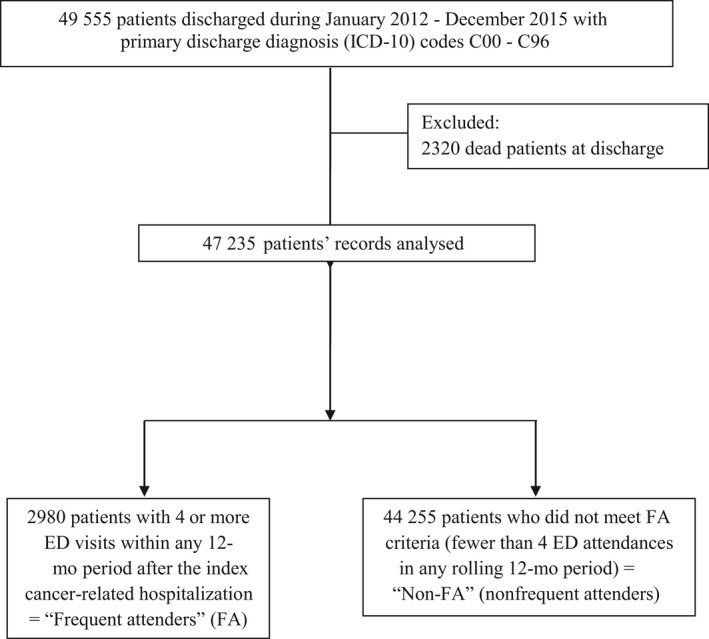
Flowchart for selection of study population

**Figure 2:**
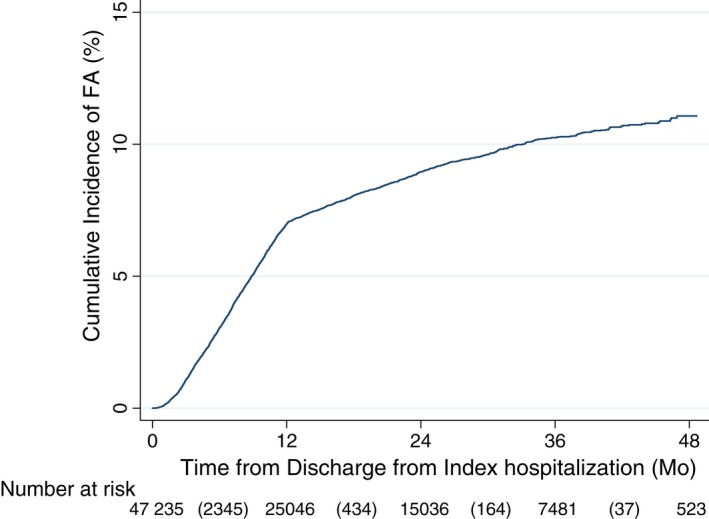
Cumulative incidence of FA, entire study population

Of the 47 235 cancer patients identified, 2980 became FAs during the study period (Figure 1). The cumulative incidence rate of FA was 7.0% by 1 year postdischarge, 9.0% at 2 years, and 10.3% at 3 years (Figure 2). FA accounted for 35.4% of all ED visits made after discharge from hospitalization (Tables 1 and 2). A higher proportion of FA patients who died during the study period died in the acute hospital (53.8%) compared to non‐FA patients (44.5%; *P* < 0.001).

**Table 1:** Patient characteristics—demographics, admission characteristics, comorbidities

	Total		Nonfrequent attenders		Frequent attenders	
	No	%	No	%	No	%
Total	47 235	100.0	44 255	100.0	2980	100.0
Demographics						
Gender						
Male	22 703	48.1	20 924	47.3	1779	59.7
Female	24 532	51.9	23 331	52.7	1201	40.3
Age, years						
Age < 17	821	1.7	709	1.6	112	3.8
17‐34	1825	3.9	1758	4.0	67	2.2
35‐44	3156	6.7	3048	6.9	108	3.6
45‐54	7315	15.5	6973	15.8	342	11.5
55‐64	12 332	26.1	11 586	26.2	746	25.0
65‐74	11 557	24.5	10 787	24.4	770	25.8
75‐84	7853	16.6	7211	16.3	642	21.5
85 and above	2376	5.0	2183	4.9	193	6.5
Median (IQR)	63 (53‐73)		62 (52‐72)		65 (56‐75)	
Ethnicity						
Chinese	33 320	70.5	31 095	70.3	2225	74.7
Indian	2029	4.3	1822	4.1	207	7.0
Malay	3893	8.2	3523	8.0	370	12.4
Other	2022	4.3	1882	4.3	140	4.7
Missing	5971	12.6	5933	13.4	38	1.3
Socioeconomic						
Medifund use						
Yes	3218	6.813	2909	6.6	309	10.4
Income percentile						
>50th percentile	32 293	68.4	30 413	68.7	1880	63.1
20th‐50th percentile	2868	6.1	2690	6.1	178	6.0
<20th percentile	12 074	25.6	11 152	25.2	922	30.9
Housing subsidy						
One‐ or two‐room housing development board (HDB) apartments	1861	3.9	1645	3.7	216	7.3
Three‐room HDB	10 334	21.9	9526	21.5	808	27.1
Four‐room HDB	12 931	27.4	11 972	27.1	959	32.2
Five‐room or Executive HDB	9342	19.8	8758	19.8	584	19.6
Private housing (condominium/landed)	5618	11.9	5316	12.0	302	10.1
Missing	7149	15.1	7038	15.9	111	3.7
Charlson comorbidity index (CCI)						
CCI = 0	29 001	61.4	27 803	62.8	1198	40.2
CCI = 1	8125	17.2	7527	17.0	598	20.1
CCI = 2	3475	7.4	3135	7.	340	11.4
CCI = 3	2326	4.9	2096	4.7	230	7.7
CCI ≥ 4	4308	9.1	3694	8.4	614	20.6
Median (IQR)	0 (0‐1)		0 (0‐1)		1 (0‐3)	
Index admission						
Length of stay (IQR)/d	5 (2‐9)		6 (2‐11)		5 (2‐9)	
Discharged	42 328	89.6	39 664	89.6	2664	89.4
Transferred	2617	5.5	2447	5.5	170	5.7
Others	2290	4.9	2144	4.8	146	4.9
ED visits (after index admission)						
Total visits made	56 532	100.0	36 516	100.0	20 016	100.0
Weekend visit	8213	14.5	5394	14.8	2819	14.1
Weekday visit	48 319	85.5	31 122	85.2	17 197	85.9
Median ED visits per patient (IQR)	0(0‐2)		0(0‐1)		6(4‐8)	
Death						
Deaths during study period	15 306		13 411		1895	
Death in acute hospital	6985	45.6	5966	44.5	1019	53.8

**Table 2:** Patient characteristics—cancer sites

Primary and secondary sites	Total		Nonfrequent attenders		Frequent attenders	
	No	%	No	%	No	%
Total	47 235	100.0	44 255	100.0	2980	100.0
Primary sites						
Other head and neck C00, C03, C04, C05, C06, C14	213	0.5	197	0.5	16	0.5
Tongue C01, C02	346	0.7	318	0.7	28	0.9
Oropharynx C9, C10	97	0.2	84	0.2	13	0.4
Nasopharynx C11	591	1.3	526	1.2	65	2.2
Hypopharynx C12, C13	103	0.2	87	0.2	16	0.5
Esophagus C15	441	0.9	378	0.9	63	2.1
Stomach C16	1894	4.0	1724	3.9	170	5.7
Small intestine C17	168	0.4	157	0.4	11	0.4
Colon C18	4767	10.1	4464	10.1	303	10.2
Rectosigmoid C19	1071	2.3	993	2.2	78	2.6
Rectum C20	1666	3.5	1540	3.5	126	4.2
Anus C21	119	0.3	107	0.2	12	0.4
Liver C22	3264	6.9	3012	6.8	252	8.5
Biliary C23, C24	510	1.1	469	1.1	41	1.4
Pancreas C25	1306	2.8	1201	2.7	105	3.5
Other facial C30, C31, C69	204	0.4	192	0.4	12	0.4
Larynx C32	356	0.8	326	0.7	30	1.0
Trachea and lung C33, C34	5447	11.5	5041	11.4	406	13.6
Thymus, heart, mediastinum C37, C38	195	0.4	187	0.4	8	0.3
Bone C40, C41	224	0.5	208	0.5	16	0.5
Skin C43, C44, C46	703	1.5	668	1.5	35	1.2
Mesothelioma, pleural C45, C384	126	0.3	107	0.2	19	0.6
Other soft tissue sarcoma C47, C49	398	0.8	375	0.9	23	0.8
Retroperitoneum C48	202	0.4	191	0.4	11	0.4
Breast C50	7013	14.9	6761	15.3	252	8.5
Female genital C51, C52, C57.7, C57.8‐9	161	0.3	150	0.3	11	0.4
Cervix C53	989	2.1	957	2.2	32	1.1
Uterus C54, C55	1670	3.5	1632	3.7	38	1.3
Ovary C56, C57.0, C57.1, C57.2, C57.3, C57.4	1379	2.9	1331	3.0	48	1.6
Male genital C60, C62, C63	257	0.5	245	0.6	12	0.4
Prostate C61	1915	4.1	1797	4.1	118	4.0
Kidney and ureter C64, C65, C66, C68	1589	3.4	1468	3.3	121	4.1
Bladder C67	1179	2.5	1073	2.4	106	3.6
Brain, spine C70, C71, C72	668	1.4	598	1.4	70	2.4
Thyroid C73	1698	3.6	1673	3.8	25	0.8
Adrenal and endocrine C74, C75	100	0.2	84	0.2	16	0.5
Other miscellaneous malignancies C7, C8, C26, C58, C76	329	0.7	314	0.7	15	0.5
Unspecified site C80	718	1.5	657	1.5	61	2.1
Hodgkin's disease C81	169	0.4	163	0.4	6	0.2
Follicular non‐Hodgkin's lymphoma (nodular) C82	201	0.4	188	0.4	13	0.4
Diffuse non‐Hodgkin's lymphoma C83	1106	2.3	1024	2.3	82	2.8
Peripheral and cutaneous T‐cell lymphomas C84	183	0.4	171	0.4	12	0.408
Other and unspecified types of non‐Hodgkin's lymphoma C85	446	0.9	420	1.0	26	0.9
Miscellaneous immunoproliferative diseases C88, C93, C94, C95, C96	142	0.3	128	0.3	14	0.5
Multiple myeloma and malignant plasma cell neoplasms C90	471	1.0	437	1.0	34	1.1
Lymphoid leukemia C91	559	1.2	503	1.1	56	1.9
Myeloid leukemia C92	756	1.6	723	1.6	33	1.1
More than one primary site	1378	2.9	1267	2.9	111	3.7
Secondary sites						
Lymph node metastases C77	9013	19.1	8485	19.2	528	17.7
Lung metastases C780, C781, C782, C783	4966	10.5	4627	10.5	339	11.4
Gastrointestinal metastases C784, C785, C786, C788	2553	5.4	2408	5.4	145	4.9
Liver metastases C787	4005	8.5	3747	8.5	258	8.7
Bone metastases C795	3843	8.1	3564	8.1	279	9.4
Brain metastases C793	1618	3.4	1494	3.4	124	4.2
Other metastases C792, C794, C796, C797, C798, C790, C791	1991	4.2	1868	4.2	123	4.1
More than one secondary site	6172	13.1	5753	13.0	419	14.1

### 3.1 Risk factors for FA

These factors were associated with an increased risk of FA: age (<17 years, hazard ratio [HR] 2.92, 95% CI 2.28‐3.74; 75‐84 years, HR 1.29, 95% CI 1.16‐1.45; and >85 years, HR 1.71, 95% CI 1.45‐2.02, relative to age 55‐64), male gender (HR 1.26, 95% CI 1.16‐1.37), Charlson comorbidity index (HR 1.21, 95% CI 1.19‐1.23), and socioeconomic factors (Medifund use, HR 1.40, 95% CI 1.23‐1.59; housing subsidy type, HR 2.12, 95% CI 1.77‐2.54).

**Figure 3:**
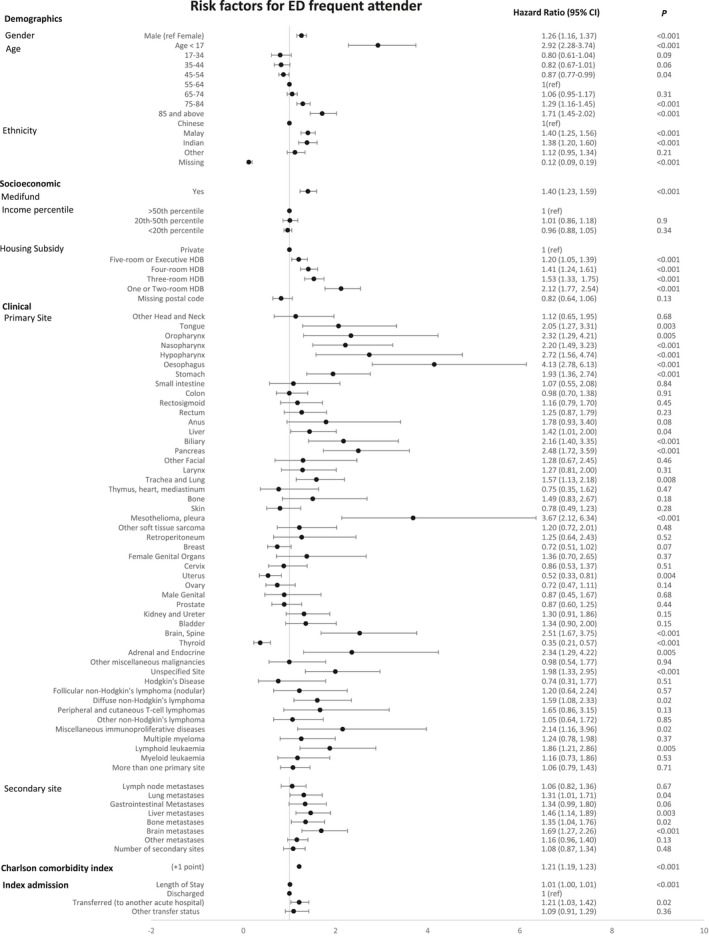
Risk factors for ED frequent attender

The following primary sites were risk factors for FA: brain and spine (HR 2.51, 95% CI 1.67‐3.75), head and neck cancers (tongue, HR 2.05, 95% CI 1.27‐3.31; oropharynx, HR 2.32, 95% CI 1.29‐4.21; hypopharynx, HR 2.72, 95% CI 1.56‐4.74), lung (trachea and lung, HR 1.57, 95% CI 1.13‐2.18; pleural, HR 3.69, 95% CI 2.12‐6.34), upper gastrointestinal (stomach, HR 1.93, 95% CI 1.26‐2.74; esophagus, HR 4.13, 95% CI 2.78‐6.13), hepato‐pancreato‐biliary (liver, HR 1.42, 95% CI 1.01‐2.00; biliary, HR 2.16, 95% CI 1.40‐3.35; pancreas, HR 2.48, 95% CI 1.72‐3.59), certain hematological malignancies (diffuse non‐Hodgkin's lymphoma, HR 1.59, 95% CI 1.08‐2.33; lymphoid leukemia, HR 1.86, 95% CI 1.21‐2.86; miscellaneous hematological malignancies, HR 2.14, 95% CI 1.16‐3.96), and unknown primary (HR 1.98, 95% CI 1.33‐2.95). Patients with thyroid (HR 0.35, 95% CI 0.21‐0.57) and uterine (HR 0.52, 95% CI 0.33‐0.81) primary sites were less likely to become FA. Among the secondary sites, metastases to brain (HR 1.69, 95% CI 1.27‐2.26), lung (HR 1.31, 95% CI 1.01‐1.71), liver (HR 1.46, 95% CI 1.14‐1.89), and bone (HR 1.35, 95% CI 1.04‐1.76) increased the risk of becoming FA (Figure 3).

**Figure 4:**
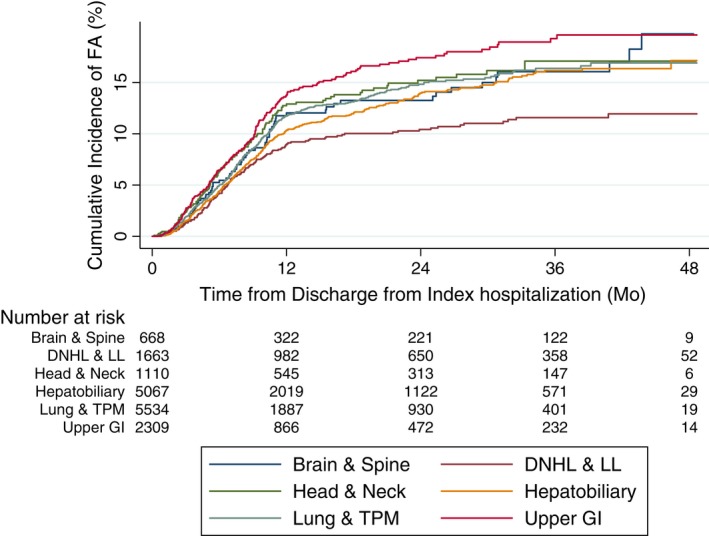
Cumulative incidence of FA, by groups of high‐risk primary sites: (a) Brain and spine (brain and spine). (b) Diffuse non‐Hodgkin's lymphoma and lymphoid leukemia (DNHL and LL). (c) Nasopharynx, base of tongue, other parts of tongue, tonsil, oropharynx, nasopharynx, piriform sinus, hypopharynx (head and neck). d. Liver, pancreatic, and biliary (hepatobiliary). (e) Lung, trachea, pleural and mesothelioma (lung and TPM). (f) Esophageal and gastric (upper GI)

When the high‐risk known primary sites were further grouped into six related groups (brain and spine; esophageal and gastric; liver, pancreatic, and biliary; lung, trachea, pleural, and mesothelioma; head and neck; and diffuse non‐Hodgkin's lymphoma and lymphoid leukemia), increase in the cumulative incidence of FA rates among patients with solid tumors occurred steadily over the first 3 years postdischarge from index hospitalization, whereas those of patients with hematological malignancies occurred mainly in the first year (Figure 4).

In the sensitivity analysis model incorporating socioeconomic factors and ethnicity, interaction effects were seen between ethnicity and housing type and between income percentile and housing type. While socioeconomic variables remained significant risk factors for FA, there was no longer a significant association between ethnicity and risk of FA. This suggested that the ethnic differences in time to FA were reflecting differences in socioeconomic status of patients.

In the model in which patients aged <17 years were excluded, patients with gastrointestinal metastases more likely to become FA. In the model including interaction terms between age‐groups and the cancer types known to be more common in pediatric and young adults, the interaction term between age less than 17 and the brain and spine primary tumors was significant. In the model using individual ICD‐10‐AM cancer sites (without the SEER regrouping), breast (C50) and ovarian cancer (C56) patients were less likely to become FA. All other risk factors remained unchanged in the sensitivity analyses.

### 3.2 Trajectory after becoming FA

**Figure 5:**
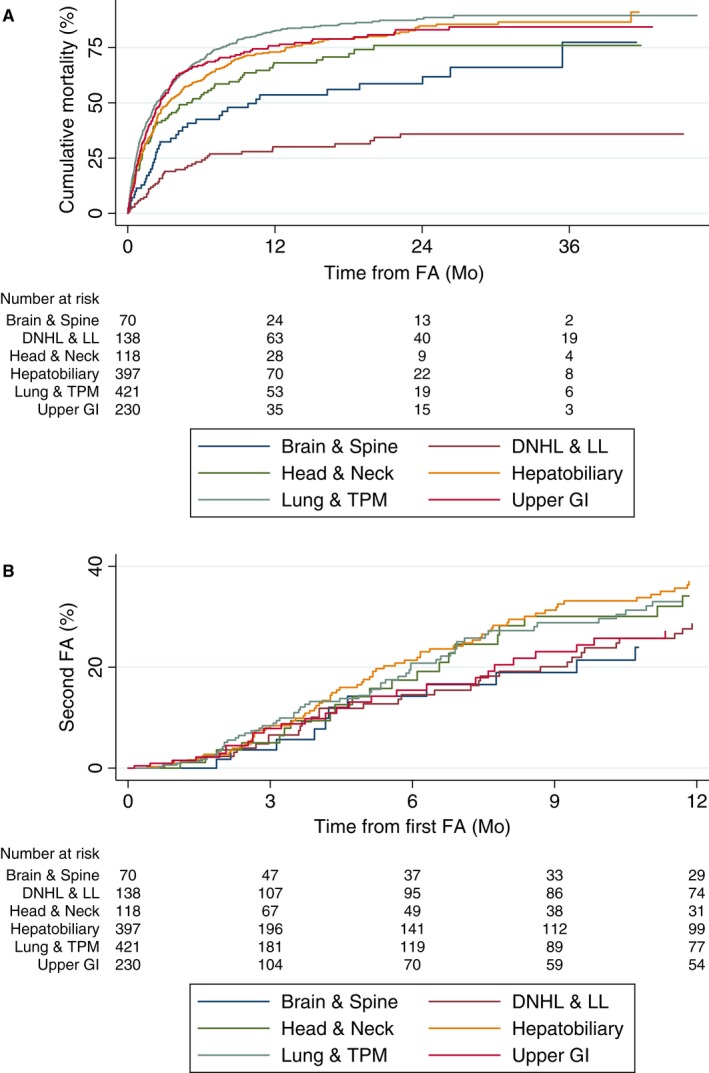
A, Cumulative mortality after FA (%), by groups of high‐risk primary sites: (a) Brain and spine (brain and spine). (b) Diffuse non‐Hodgkin's lymphoma and lymphoid leukemia (DNHL and LL). (c) Nasopharynx, base of tongue, other parts of tongue, tonsil, oropharynx, nasopharynx, piriform sinus, hypopharynx (head and neck). (d) Liver, pancreatic, and biliary (hepatobiliary). (e) Lung, trachea, pleural, and mesothelioma (Lung and TPM). (f) Esophageal and gastric (upper GI). B, Cumulative incidence of second FA (%), by groups of high‐risk primary sites: (a) Brain and spine (brain and spine). (b) Diffuse non‐Hodgkin's lymphoma and lymphoid leukemia (DNHL and LL). (c) Nasopharynx, base of tongue, other parts of tongue, tonsil, oropharynx, nasopharynx, piriform sinus, hypopharynx (head and neck). (d) Liver, pancreatic, and biliary (hepatobiliary). (e) Lung, trachea, pleural, and mesothelioma (Lung and TPM). (f) Esophageal and gastric (upper GI)

Of the 2980 FA, 39.2% died within 3 months of becoming FA. The 6‐month and 12‐month cumulative mortality rates of FA were 51.5% and 62.5%, respectively. Of the six high‐risk primary site groups, diffuse non‐Hodgkin's lymphoma and lymphoid leukemia patients had the highest 12‐month survival rate of 69.9%, followed by brain and spine patients at 46.4%. The remaining solid tumor patients had a 12‐month survival rate of 17.6%‐31.9% (Figure 5A).

Over 40% of FA patients made another four or more ED visits within the 12‐month period after becoming FA. The hepatobiliary group had the highest 12‐month cumulative incidence rates of repeat FA (Figure 5B).

## 4 DISCUSSION

Despite being heavy users of ED services, specific factors that put cancer patients at risk of becoming FA have yet to be defined. In this study, we found that age, male gender, comorbidities, socioeconomic factors, and certain cancer sites were associated with higher risk of FA. Patients admitted with cancer sites associated with better prognoses (breast, thyroid, uterine, or ovarian primaries) were less likely to become FA. This result is consistent with other ED studies showing proportionately fewer breast cancer patients.^1^, ^9^ Longer length of stay of index hospitalization and the need for transfer to another acute hospital likely reflected the complexity of treatment required, although social factors could also affect length of stay.

To our knowledge, this study is the first to examine ED frequent attendance by cancer patients as a whole and to identify disease‐specific risk factors for high ED utilization. Most studies have focused on specific cancer subpopulations, such as end‐of‐life patients. A recent meta‐analysis of ED utilization in end‐of‐life cancer patients found that male gender, ethnic minority, low socioeconomic status, and lung cancer were independent risk factors for increased ED utilization.^7^ Our study revealed additional risk factors associated with FA. We included patients that had recently undergone curative oncologic treatment, rather than limiting the analyses to end‐of‐life patients. Furthermore, given the cohort size and diversity of diagnoses, we were able to divide the malignancies by specific diagnoses rather than collective groups such as “all gastrointestinal” cancers.^5^, ^25^ This could account for the positive correlation between a diagnosis of esophageal, stomach, liver, pancreatic or biliary cancer and FA, as each of these was analyzed independently of neutral‐risk gastrointestinal cancers, such as colorectal cancer patients, which account for the largest group of gastrointestinal cancers in most populations. Another study has also reported a high proportion of head and neck cancer patients utilizing ED,^5^ similar to the findings in our study, but again, this was in the end‐of‐life setting.

Many smoking‐associated cancers (lung, esophageal, head and neck) were associated with FA. The increased risk of FA for these patients could be compounded by the burden of smoking‐related noncancer comorbidities, concordant with the finding that Charlson comorbidity index was significant in our model. Lung cancer, known to be a resource‐intensive cancer primary site,^26^, ^27^ was the most common cancer in the FA group. The less common brain and spine primary sites would likely cause a rapid decline in independence, even before patients succumb to their disease. Our study found that the risk of FA was even higher for several other primary malignancies (liver, pancreatic, biliary, esophageal, stomach, brain and spine, head and neck). One commonality for several of the FA risk cancer primaries (esophageal, gastric, liver, pancreatic, biliary, lung, trachea, pleural, head and neck cancers) is the requirement for tubes, stents, or other paraphernalia that can be dislodged, blocked, or have a number of technical issues not easily managed in the community. These include biliary stents for hepatobiliary sepsis, endoscopic feeding tubes for upper gastrointestinal blockage, pleural drainage tubes for lung effusions, tracheostomy, and feeding tubes for the head and neck cancers. We plan to focus our future analyses on the contribution of these paraphernalia to ED visits due to blockage or displacement, and examine the chronologic relationship between ED attendances and cancer‐specific treatment.

In contrast, FA among lymphoma and leukemia patients is likely explained by infectious complications arising from myelotoxic and immunosuppressive treatment, with patients at continued risk throughout the course of treatment. This may explain the difference in the shape of cumulative incidence of FA curves for these patients, when compared to the patients with high‐risk solid tumors.

Secondary analyses of the trajectory of FA patients after they became FA yielded additional insights. The significant proportion of cancer patients who became FA before the end of life highlighted the additional ED burden and unmet needs for patients whose life expectancy would exceed qualifying for hospice care. Many end‐of‐life studies focus on ED visits in the last 2 weeks, 30 days or 6 months of life,^3^, ^6^, ^7^ or once advanced disease is diagnosed.^5^ While end‐of‐life care is an important trigger for ED visits and unplanned admission, studies focusing only on end‐of‐life ED utilization could include patients attending ED for terminal care, but whose overall ED utilization was not high. Our study shows the utility of applying definitions of high ED utilization (four or more visits in 12 months) to cancer patients regardless of survival, suggesting that specific cancer types are associated with FA.

Many FA patients became repeat FA in the 12 months after they first met FA criteria. A higher proportion of FA patients died in an acute hospital compared to non‐FA patients, possibly reflecting a higher degree of unmet needs. With the improved survival from advances in cancer treatment, and the resulting increased life expectancy for patients with advanced disease, patients with the risk factors identified in our study may benefit from additional support, regardless of life expectancy. We hope these results can inform healthcare planning needs for these high‐risk groups of patients. For example, for FA groups who have high mortality after becoming FA (upper gastrointestinal, hepatobiliary, head and neck, lung), it is likely that high intensity and earlier introduction of end‐of‐life care would be helpful. In contrast, for FA groups who have high repeat FA within the subsequent 12 months, and yet have reasonable survival (hematological malignancies, brain and spine), alternative support measures may be needed.

We believe that the data presented here can be translated to First World countries. Singapore is an urban country with long life expectancy and a well‐developed healthcare system. Access to a nationwide billing database allowed accurate identification and definition for the cohort examined, and hence the large sample size and the good quality of data linkage. We found a low proportion of deaths in ED, probably reflecting good access to hospice services.^28^

Some aggressive cancers (eg, melanoma) common in Western populations were rare in our study population, and hence, the numbers in our study could be too low to manifest FA risk factors for these cancers. In addition, the low numbers of pediatric and young adult patients in the study (as expected of a small country with an aging population and low birth rate), make it difficult to further stratify at‐risk age‐groups for pediatric and young adult tumors. Hence, it would be good for researchers with access to data with higher numbers of pediatric and young adult patients to focus on these tumors in future studies. Nevertheless, our findings should be generalizable for the cancers positively associated with FA in our study.

One limitation was that we could not link our data to the various independent home hospice organizations in Singapore; hence, we could not examine the effect of timely referral and frequency of home hospice visits on ED utilization, shown to reduce ED utilization.^4^, ^29^, ^30^ The majority of dying cancer patients in Singapore receive community hospice services,^31^ and the high‐risk groups we found in our study may benefit from the early referral.

## 5 CONCLUSIONS

There are both clinical and socioeconomic risk factors for FA suffering from cancer, and the high‐risk groups of cancer patients identified in this study may benefit from targeted models of care. These findings provide an important framework to institute the necessary multidisciplinary support structures to prevent these attendances. Improving care coordination and expansion of existing community resources to support these patient groups should be considered when planning emergency and oncologic services, as this is clearly an urgent unmet clinical need.

## CONFLICT OF INTEREST

None declared.
