# Supplementary material for: Cancer patients as frequent attenders in emergency departments: A national cohort study
Source: Cancer Med. 2018 Aug 17;7(9):4434–46. doi: 10.1002/cam4.1728 (PMC6144141; doi:10.1002/cam4.1728)
Supplement: Supplementary file 1 [file CAM4-7-4434-s001.docx]

**Supplementary Table**

Primary cancer sites with low event rates (fewer than ten patients becoming FA over the study period) were re-grouped as follows:

| Low-Event Rate Groups | Sites |
| --- | --- |
| Other Head and Neck  C00 C03 C04 C05 C06 C14 | Lip, gum, floor of mouth, palate, other parts of the mouth, and other sites in lip, oral and pharynx |
| Other Facial C30 C31 C69 | Nasal cavity, middle ear, accessory sinuses, grouped with eye and adnexa |
| Bone C40 C41 | Bone and articular cartilage of limbs, bone and articular of other and unspecified sites |
| Skin C43 C44 C46 | Malignant melanoma of skin, other malignant neoplasms of skin, Kaposi’s sarcoma |
| Other soft tissue sarcoma C47 C49 | Peripheral nerves and autonomic nervous system, other soft tissue sarcoma |
| Brain, Spine C70 C71 C72 | Brain, meninges and spinal cord |
| Other miscellaneous malignancies C7 C8 C26 C58 C76 | Parotid gland, other and unspecified major salivary glands, other and ill-defined digestive organs, placenta, choriocarcinoma, chorionepithelioma, other and ill-defined sites |
| Miscellaneous immunoproliferative diseases C88 C93 C94 C95 C96 | Malignant immunoproliferative diseases, monocytic leukaemia, other leukaemias of specified cell type, leukaemia of unspecified cell type, Letterer-Siwe disease, malignant histiocytosis, malignant mast cell tumour; malignant mastocytosis, true histiocytic lymphoma, other specified malignant neoplasms of lymphoid, hematopoietic and related tissue, malignant neoplasm of lymphoid, hematopoietic and related tissue, unspecified |
